# Glaucoma diagnosis using multi-feature analysis and a deep learning technique

**DOI:** 10.1038/s41598-022-12147-y

**Published:** 2022-05-16

**Authors:** Nahida Akter, John Fletcher, Stuart Perry, Matthew P. Simunovic, Nancy Briggs, Maitreyee Roy

**Affiliations:** 1 School of Optometry and Vision Science, UNSW Sydney, Sydney, NSW 2052 Australia; 2 School of Electrical Engineering and Telecommunications, UNSW Sydney, Sydney, NSW 2052 Australia; 3 School of Electrical and Data Engineering, University of Technology Sydney, Sydney, NSW 2007 Australia; 4 Save Sight Institute, The University of Sydney, Sydney, NSW 2006 Australia; 5 Sydney Eye Hospital, Sydney, NSW 2000 Australia

**Keywords:** Machine learning, Biomedical engineering

## Abstract

In this study, we aimed to facilitate the current diagnostic assessment of glaucoma by analyzing multiple features and introducing a new cross-sectional optic nerve head (ONH) feature from optical coherence tomography (OCT) images. The data (n = 100 for both glaucoma and control) were collected based on structural, functional, demographic and risk factors. The features were statistically analyzed, and the most significant four features were used to train machine learning (ML) algorithms. Two ML algorithms: deep learning (DL) and logistic regression (LR) were compared in terms of the classification accuracy for automated glaucoma detection. The performance of the ML models was evaluated on unseen test data, n = 55. An image segmentation pilot study was then performed on cross-sectional OCT scans. The ONH cup area was extracted, analyzed, and a new DL model was trained for glaucoma prediction. The DL model was estimated using five-fold cross-validation and compared with two pre-trained models. The DL model trained from the optimal features achieved significantly higher diagnostic performance (area under the receiver operating characteristic curve (AUC) 0.98 and accuracy of 97% on validation data and 96% on test data) compared to previous studies for automated glaucoma detection. The second DL model used in the pilot study also showed promising outcomes (AUC 0.99 and accuracy of 98.6%) to detect glaucoma compared to two pre-trained models. In combination, the result of the two studies strongly suggests the four features and the cross-sectional ONH cup area trained using deep learning have a great potential for use as an initial screening tool for glaucoma which will assist clinicians in making a precise decision.

## Introduction

Glaucoma is a potentially blinding optic neuropathy with a variety of underlying etiologies characterized by the loss of retinal ganglion cells (RGCs). It is characterized clinically by anatomical changes of the optic nerve head (ONH), mainly thinning and posterior bowing of the lamina cribrosa sheets seen clinically as ONH cupping^1^. Detection and monitoring of glaucomatous optic neuropathy depend on several clinical features which are observed and assessed before making a clinical decision^2^. Currently, glaucoma diagnosis and monitoring require a complete eye examination and additional testing and gathering of a slew of data, which can be challenging to interpret. Furthermore, there is a significant overlap in the ocular features of normal subjects and patients with early glaucoma. For these reasons, there is interest in developing complementary techniques—such as artificial intelligence (AI) systems^3^—to assist in distinguishing true pathology from normal variability and true progression from inter-test variability.

Following the recent implementation of AI within ophthalmology, several machine learning (ML) algorithms have been investigated and developed for automated glaucoma detection that can quickly process the retinal images and accurately detect glaucomatous damage on pathological tests compared to conventional methods. Automated glaucoma detection using simpler ML to advanced deep learning (DL) algorithms, mainly from ocular images, has been widely researched with variable outcomes. Most DL algorithms trained from fundus and OCT images perform two common steps; segmentation of the region of interest and classification of glaucomatous and non-glaucomatous eyes. In the early stages, fundus photographs have been widely used to evaluate and detect glaucoma using AI techniques^4–8^. Ting et al.^9^ trained a DL model on 71,896 validated retinal fundus photographs to detect referable possible glaucoma with an AUC of 0.942.

Additionally, Asaoka et al.^10^ applied a transfer learning model to macular OCT images and evaluated its diagnostic performance on an independent dataset consisting of normal eye and early-onset glaucomatous eyes. The model's AUC was 0.93, which was significantly larger compared to other ML methods such as support vector machine (SVM) and random forest (RF). An et al.^11^ trained both fundus and OCT images using the VGG19 model to distinguish glaucomatous from normal eyes and achieved an AUC of 0.94 for fundus and an AUC of 0.94 for four features of OCT images, and the combination of all images achieved an AUC of 0.96.

Furthermore, Devalla et al.^12^ investigated the ability of a dilated-residual U-Net (DRUNET), a deep learning network, to detect glaucoma on 100 OCT B-scan images. DRUNET achieved a mean sensitivity of 92% and specificity of 99% in detecting both healthy and glaucomatous eyes. In another study, Gomez-Valverde et al.^13^ trained five convolutional neural network (CNN) models-standard CNN, VGG19, ResNet50, GoogleNet and DENet using the RIM-ONE dataset and VGG19 performed best for glaucoma detection with an AUC of 0.94, the sensitivity of 87.0% and specificity of 89.0%. Asaoka et al.^14^ used a local dataset consisting of 1364 glaucoma and 1768 healthy retinal images to train from a pre-trained model ResNet and achieved an AUC of 0.95.

A small number of studies have used visual field (VF) data to train DL algorithms to detect glaucomatous damage, and those that exist show a similar, sometimes better, performance comparative to glaucoma experts^15–17^. Li et al.^18^ trained a deep CNN to differentiate between glaucomatous and non-glaucomatous VFs, with probability deviation (PD) maps as the data input. It achieved an AUC of 0.96 with accuracy, sensitivity, and specificity of 87.6%, 93.2% and 82.6%, respectively. Bizios et al.^19^ showed that a feed-forward, multilayered artificial neural network (ANN) had considerably better performance and diagnostic accuracy in distinguishing between normal and glaucomatous VFs compared to conventional STATPAC global indices [Glaucoma Hemifield Test (GHT) and pattern standard deviation (PSD)] and achieved an AUC of 0.98. Kucur et al.^20^ have also developed a CNN capable of discriminating between normal and early glaucomatous VFs with an average precision score of 87%.

There is now compelling evidence that training a ML classifier model with combined structural and functional features could enhance discriminatory power compared to models trained with either structure or function alone^21^. For instance, clinical factors, intraocular pressure (IOP), and corneal thickness per se have been shown to partially enrich the diagnostic accuracy of the algorithms^22^. Brigatti et al.^23^ used a neural network to train combined features from standard automatic perimetry (SAP) indices (mean defect, corrected loss variance, and short-term fluctuation) and structural data (cup/disk ratio, rim area, cup volume, and nerve fiber layer height) together from a total of 185 glaucoma and 54 normal subjects and achieved 90% sensitivity and 84% specificity. Bowd et al.^24^ added retinal nerve fiber layer (RNFL) thickness and SAP parameters using a relevance vector machine classifier and achieved an AUC of 0.85. In another study, Grewal et al.^25^ used an ANN model to detect glaucoma combining age, sex, myopia, IOP, ONH, and RNFL, SAP parameters and achieved an AUC of 0.77. In a recent study, Kim et al.^22^ trained four ML algorithms: C5.0, RF, SVM, and k-nearest neighbors (KNN) combining Age, IOP, corneal thickness, RNFL, GHT, mean deviation (MD), PSD from total 342 subjects. The RF model performed best among the four models with an AUC of 0.98.

However, as listed in the literature, a few studies have been done for glaucoma detection using both machine and deep learning techniques combining structural and functional features. Moreover, as glaucoma is a multifactorial disease and is challenging to detect early on; hence, clinicians also consider risk factors. Such features include older age, family history of glaucoma, gender and ethnicity (e.g., African Americans are at higher risk of open-angle glaucoma)^26^. Higher IOP, decreased central corneal thickness (CCT), and myopia are other established risk factors for glaucoma^27,28^. We could not find any ML studies that used combined features including risk factors for early glaucoma detection. Moreover, feature optimization from maximum clinical input is now highly requisite for both clinicians and glaucoma patients. Besides, this will resolve the time and resource limitations of AI models. Given the handful of proposed techniques, it is warranted to develop an effective AI algorithm that combines entire patient history with as much real-world data as possible, which can exceed human performance in diagnosing glaucoma.

Thus, we aimed to explore and compare the optimal features for diagnosing glaucoma by combining functional, structural, and demographic/historical risk factor data. Our initial research aims to identify significant features aiding the detection of glaucomatous changes and observe the classification performance using machine learning techniques trained from the optimized features. Besides, in our study, we included the majority of the glaucoma patients from the early group, so that the ML model able to detect glaucoma at early stages. In addition to using the 2D data, we also used a new cross-sectional ONH OCT image which can be added as a new clinical feature for diagnosing glaucoma and enhance the accuracy if combined with 2D data.

## Methods

### Datasets

Clinical data from two subject groups examined between 2015 and 2018 at the Centre for Eye Health, UNSW Sydney were analyzed (*n* = 200, consisting of 100 normal subjects and 100 glaucoma patients). Normal patients were matched to cases on age group (30–39, 40–49, 50–59, 60–69 and 70–85). Our main aim was to optimize the features that can also help to diagnose early glaucoma. Therefore, the glaucoma data consists of 73 early, 21 moderate, 4 advanced and 2 severe patients. The glaucoma stage labelling was done according to the criteria of Mills et al.^29^. Moreover, unseen data from 55 patients consisting of 25 glaucomatous and 30 normal eyes have been used as a test dataset.

Because glaucoma is considered a complex eye disease, optimizing its detection and monitoring is an important public health issue. To this aim, we have explored optimal features, including functional, structural, demographic findings, and known risk factors for glaucoma. Table 1 summarizes the possible features of primary open-angle glaucoma (POAG)^30–33^.

**Table 1:** The possible primary open-angle glaucoma diagnosis features.

Structural features	Functional features	Demographic features/clinical risk factors
Optic nerve head damage	Mean deviation (MD)	Age, gender, ethnicity
Inner macular thinning	Pattern standard deviation (PSD)	Family history
Thinning of the circumpapillary retinal nerve fiber layer (cpRNFL)	Visual field index (VFI)	Intraocular pressure (IOP)
Increased cup to disk ratio (CDR)		Refractive error
Central corneal thickness (CCT)		High or low blood pressure, Diabetes, previous eye injury

Based on possible glaucoma diagnosis features, we collected most of the data of Table 1 from the Cirrus HD-OCT (Carl Zeiss Meditec) and a total of 11 features (age, gender, average RNFL thickness, CDR, corneal thickness, IOP, MD, PSD, Spherical Equivalent (SE), ethnicity and family history) were extracted.

We performed a pilot study of segmentation and extraction of a new region of interest (ROI) from the ONH OCT B-scan (per eye 6 scans) images (Spectralis OCT, Heidelberg Engineering) of 60 eyes, including 30 normal and 30 glaucoma among 200 patient data. The ethics approval for the data collection was provided by the relevant ethics committee of the UNSW Sydney, and the study followed the tenets of the Declaration of Helsinki. Patients provided written informed consent for the use of their de-identified clinical data for research purposes.

### Statistical analysis

Using traditional sources of information for glaucoma diagnosis, we first calculated the independent *t* test and performed a receiver operating characteristic (ROC) curve analysis to find significant features and examined the separability of their distributions using the AUC. Moreover, we calculated the power of the sample size for both the independent *t* test and ROC curve.

### Segmentation of ONH feature

**Figure 1:**
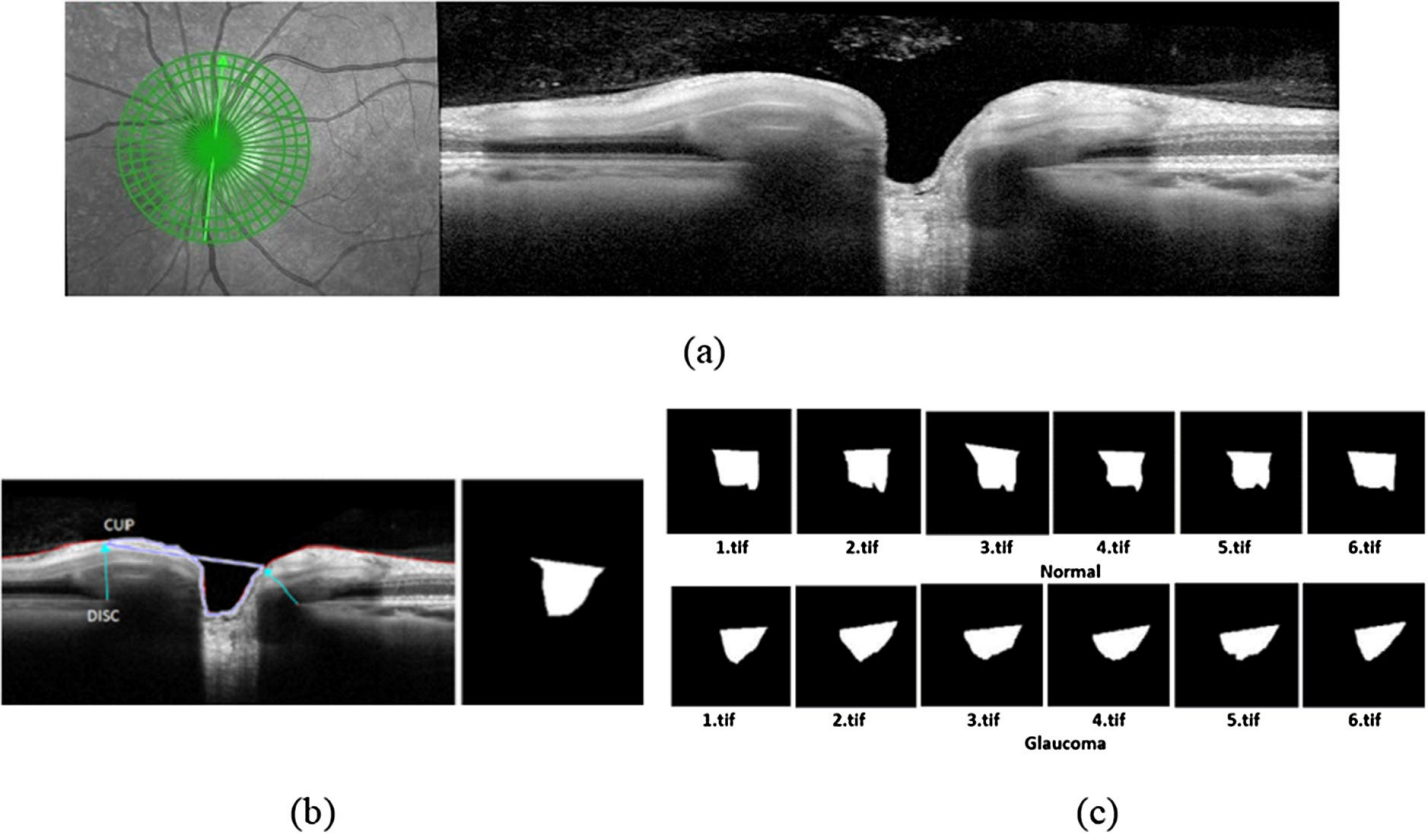
(**a**) The radial pattern of optic nerve head OCT with B-scan. (**b**) ROI selection and extraction from B-scan. (**c**) Final ROI extracted images for glaucoma and normal subjects.

A pilot study was performed to segment the cup surface area of the ONH OCT B-scan images of 60 eyes, including 30 normal and 30 glaucoma patients. For this study, the normal and glaucoma patient’s average age was 47 ± 11 and 61 ± 12.36, respectively. As glaucoma is optic neuropathy, we specifically explored features of the cross-sectional OCT B-scans of the optic nerve. The B-scans were radially arranged from the center of the ONH, as shown in Fig. 1a. We explicitly introduced a new technique for segmentation and extraction of the cup surface area from the B-scan of OCT images and calculated the mean area of the first 6 B-scans of the ONH among a total of 24 OCT scans^34^, based on knowledge regarding known anatomical changes occurring at the ONH, specifically in the superior and inferior region of the eye in glaucoma^35^. The initial point of ROI selection was measured from the disk to cup with a minimum rim width as demonstrated in Fig. 1b and the cup surface area was selected from the rim point using a freehand polygon method and a binary mask was generated from the resultant polygon. The freehand segmentation was performed in MATLAB R2019b, and the cup area, mean cup area, and SD were calculated from binary images using the open-source software [ImageJ (www.imagej.nih.gov/ij/)]. We calculated the mean area of the cup surface from the first 6 B-scans of 40 eyes among of 60. The resultant binary images are shown in Fig. 1c. Later, a DL algorithm was applied to the segmented OCT B-scan images. The segmented OCT B-scan was cropped from the original OCT scan and the new image size was 383 × 197 pixels.

### Machine learning models

The statistically significant features were trained using two machine learning algorithms, a LR and DL technique, to observe the classification accuracy of glaucoma detection. We initially started with a simple, efficient algorithm, LR, as our prediction based on a simple binary classification from the parametric dataset.

For the classification using only significant features, we divided the 200 datasets into 80% training and 20% validation data. The parameters for LR classifier were ‘libliner’, a linear classifier solver, L2 penalty (squared magnitude of coefficient), a regularization technique to reduce the overfitting problem, and the tolerance value was 0.0001. We then applied a DL model consisting of four layers to investigate whether the classification accuracy is enhanced. It is assumed that the accuracy will be increased as a single neuron in the deep neural network refers to a similar input–output mapping as occurs in LR^36^. For the DL technique, we used a sequential model consisting of four layers, 64 nodes at each layer with ReLU (Rectified Linear Units) as the activation function. For the last layer, we used one node with a sigmoid activation function that squeezes all input values to an output range between 0 and 1. The model was compiled by using Adam^37^, a momentum-based optimizer with the loss function binary_crossentropy, which supplied the output in the form of a probability. To evaluate the performance of the models, an unseen 25 glaucomatous and 30 normal eye data were used as a test dataset.

In the pilot study, a simple CNN architecture has been developed to classify eyes as normal or glaucomatous directly from the segmented OCT B-scans. To evaluate the performance of our proposed CNN model, the result has been compared with two widely used pre-trained models. A total of 360 binary segmented OCT images of 60 eyes were used to train the CNN using two subject groups: 180 images for normal and 180 from glaucoma patients. Due to the limited dataset, k-fold cross-validation was conducted for assessing the model’s generalizability on unseen data.

The cross-validation is performed k times to allow for the use of all subsets exactly once as a test set. Model performance is determined according to the average of model evaluation scores calculated across the k test subsets. We used five-fold cross-validation adapted from^38^ to utilize the whole dataset for training and observed the performance on the test dataset. The images were resized to 224 × 224 pixels during the data augmentation using MATLAB imageDataAugmenter. Hence, the rescaling will not affect the actual shape and volume of the ONH cup surface. The augmentation was performed using four arguments: ‘RandRotation’, ‘RandXTranslation’, ‘RandYTranslation’, ‘RandXReflection’ (horizontally reflected) and ‘RandXShear’ (horizontally sheared). No other pre-processing was performed.

We used a CNN architecture of 24 layers consisting of five convolutional layers (3 × 3) with increased kernel sizes 32, 64, 128, 256 and 512. We added a ReLU and batch normalization layer after every convolutional layer to accelerate and improve the initialization of the network^39,40^. Then a max-pooling (2 × 2) layer has been added for every convolutional layer following the batch normalization and ReLU layer. A fully connected layer was added at the end of the network for final classification. We also added one dropout layer before the fully connected layer to exclude 25% of neurons from the previous layer of CNN to overcome the overfitting problem during the training period^41^. For the binary classification output, we used the “softmax” function instead of “sigmoid” as the softmax output is presented as the probability of the input belonging to the associated class, whereas the “sigmoid” function simply creates an output between 0 to 1^42^. To train the network, we used Root Mean Squared Propagation (RMSProp) optimizer with an initial learning rate of 1 × 10^–4^. The training 'MiniBatchSize' was set to 12 with ‘MaxEpochs’ 20.

To evaluate the performance of our DL model, we used two pre-trained models; ResNet18^43^ and VGG16^44^, which are trained from the ImageNet database (with more than a million images)^45^. The two models are specifically used for medical image classification, segmentation, and feature extraction^46,47^. These two models exhibited comparatively better performance in the literature than other DL models on OCT images for glaucoma detection^11,13,14,47^. ResNet18 and VGG16 consist of total 71 and 41 layers, respectively, and both require 224 × 224-pixel size images for the input layer. We used five-fold cross-validation for both networks and the same data augmentation and Stochastic Gradient Descent with Momentum (SGDM) model optimizer was used. To obtain the best results for the two models, training hyperparameters: learning rate, epochs and batch sizes were tuned to achieve optimal results. A batch size of 12, 20 epochs, and a learning rate of 1 × 10^–5^ was determined for VGG16 and a batch size of twelve, 20 epochs and a learning rate of 1 × 10^–4^ was determined for ResNet18. The other hyperparameters associated with both network models were set according to their original structure.

### Grad-CAM visualization

The gradient-weighted class activation mapping (Grad-CAM) technique was used to visualize the learned features of the network which influenced the model to classify the two groups. The Grad-CAM calculates the gradient of the image score for the specific class and estimates the gradient of the final classification score relating to the weights of the last convolutional layer. The Grad-CAM generates heatmap transparently on the image, where deep red is considered as the peak value of the predicting class and deep blue is the lowest class value^48^.

## Results

### Power and sample size estimation

Considering an effect size of 0.50, α error of 0.05 and group allocation ratio of 1:1 a minimum of 88 patients in each group is required to reach a statistical power of 95%. In our study, for n = 100 in each group has greater than 95% power to detect a statistical difference using independent *t* test for continuous variables. For ROC curve analysis, n = 100 in each group has greater than 90% power to detect a ROC AUC of 0.80 for a continuous predictor assuming a null ROC AUC of 0.70. For a dichotomous predictor, power is greater than 80%.

### Classifying based on multiple features

The patient demographics are presented in Table 2. Group differences were tested using independent samples *t* test (continuous variables) or χ^2^ test (categorical variables).

**Table 2:** Demographic data of the study population.

Parameters/features	Normal (n = 100)	Glaucoma (n = 100)	*p*-value
Age (mean ± SD)	55.11 ± 11.19	55.42 ± 12.12	0.851
Gender	Female 51, Male 49	Female 31, Male 69	0.004
Family history	Yes: 22, N/A:78	Yes: 22, N/A: 78	1
Ethnicity	Asian-27, Caucasian-62, Indigenous Australian-1 Hispanic (Central and South American)-2, Indian-2, Arab Caucasoid (Middle eastern)-3, African-1, Unknown-2	Asian-41, Caucasian-35, Indigenous Australian-2 Hispanic (Central and South American)-2, Indian-5, Arab Caucasoid (Middle Eastern)-3, African-3, Pacific Islander-1, Unknown-7, Refused-1	N/A
SE (mean ± SD)	− 0.75 ± 2.17	− 1.33 ± 2.58	0.057
Average RNFL thickness (mean ± SD)	94.75 ± 8.97	77.62 ± 10.76	< 0.001
CDR (mean ± SD)	0.58 ± 0.15	0.72 ± 0.12	< 0.001
Corneal thickness (mean ± SD)	567.56 ± 37.16	557.56 ± 32.92	0.045
IOP (mean ± SD)	15.9 ± 3.12	17.87 ± 4.40	0.001
MD (mean ± SD)	− 0.82 ± 2.21	− 3.20 ± 5.01	< 0.001
PSD (mean ± SD)	2.03 ± 1.52	3.51 ± 2.52	< 0.001

**Figure 2:**
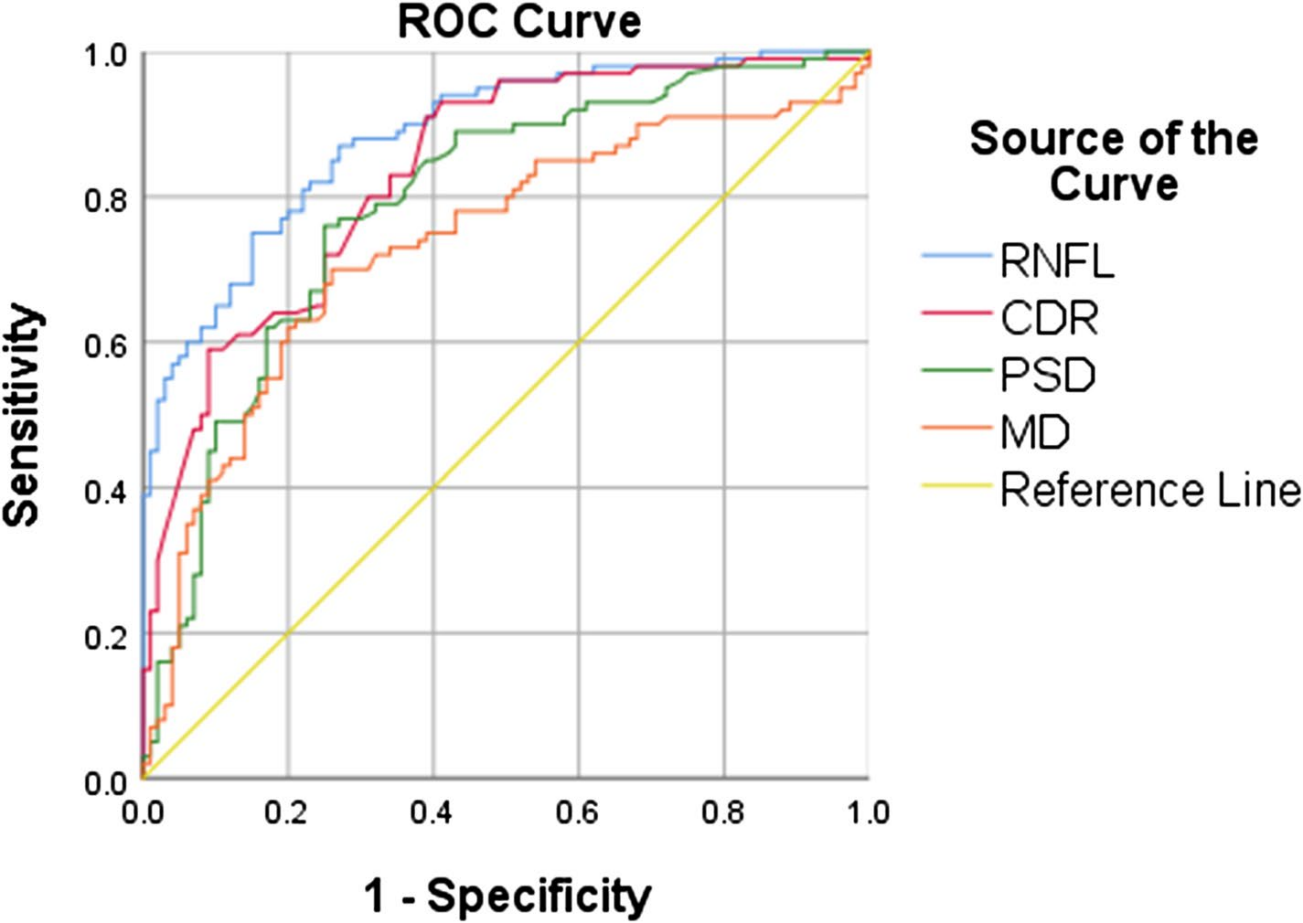
ROC curve for the features (AUC) > = 0.7

Comparison of the extracted features in two groups suggests that only RNFL, CDR, IOP, MD and PSD are statistically significant (p < 0.05). Furthermore, the AUC of the ROC was > 0.7 for RNFL, CDR, PSD and MD (Table 3 and Fig. 2), and IOP had a poor separability between the two groups (AUC 0.63).

**Table 3:** The area under the ROC curve for all features. Significant values are in bold.

Features	AUC	Std. error
RNFL_thickness	**0.89**	0.02
CDR	**0.84**	0.02
PSD	**0.79**	0.03
MD	**0.74**	0.04
IOP	0.63	0.04
Gender	0.6	0.04
Family_history	0.5	0.04
SE	0.43	0.04
Corneal_thickness	0.44	0.04

### Classification results of LR and DL

For glaucoma classification, we trained the LR and DL algorithms with the four features MD, PSD, RNFL and CDR; and observed the performance on both validation and unseen test dataset. We achieved the best performances for the LR classifier with 100 iterations, and the DL classifier with 1000 epochs and a batch size of 20. To evaluate both models’ performance, AUC was calculated from the ROC curve comparing true positive rate vs false positive rate, and sensitivity/specificity ratios were calculated from the confusion matrix as shown in Table 4. Every confusion matrix provides four outcomes—true positive, true negative, false positive and false negative. In our case:

**Table 4:** Confusion matrix of (a) LR from the result of validation dataset (b) DL from the result of the validation dataset (c) test dataset (similar for LR and DL).

		Actual values	
		Glaucoma	Normal
**(a)**			
Predicted values	Glaucoma	17 (TP)	2 (FP)
	Normal	0 (FN)	21 (TN)
**(b)**			
Predicted values	Glaucoma	17 (TP)	1 (FP)
	Normal	0 (FN)	22 (TN)
**(c)**			
Predicted values	Glaucoma	25 (TP)	2 (FP)
	Normal	0 (FN)	28 (TN)

1. True positive (TP): correct glaucoma prediction
2. False-positive (FP): incorrect glaucoma prediction
3. True negative (TN): correct normal prediction
4. False-negative (FN): incorrect normal prediction

We define sensitivity or true positive rate TPR = TP/(TP + FN) and

Specificity, or true negative rate TNR = TN/(TN + FP)^49^.

From the confusion matrix of Tables 4 and 5, we can see both LR and DL models can successfully classify the group with an accuracy of 95% and 97%, respectively, with the four features. The DL model with an AUC of 0.98, a sensitivity of 100%, and a specificity of 96% performs better than the LR model. However, the LR and DL models showed similar performance on the test dataset with an accuracy of 96%, sensitivity of 100%, and a specificity of 93%.

**Table 5:** Performance summary of the LR and DL models on validation data, trained from the significant features of glaucoma.

Classifier	AUC	Sensitivity (%)	Specificity (%)	Accuracy (%)
LR	0.97	100	91	95
DL	0.98	100	96	97

### DL results from segmented images

**Figure 3:**
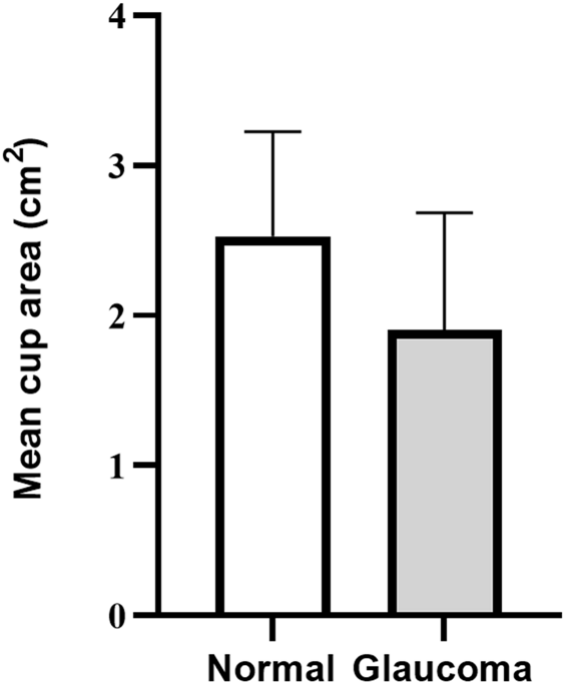
The mean cup surface area of the first 6 OCT B-scans (cross-sectional) of the ONH for normal and glaucoma groups.

We calculated the mean cup surface area from the segmented cross-sectional OCT B-scans and the mean cup area found significant [p < 0.05 (p = 0.01)] for two groups and to be lower for glaucoma than the normal group as shown in Fig. 3.

The results of our proposed DL model trained from segmented images are shown below and compared with the two pre-trained models using a confusion matrix in Tables 6, and 7.

**Table 6:**
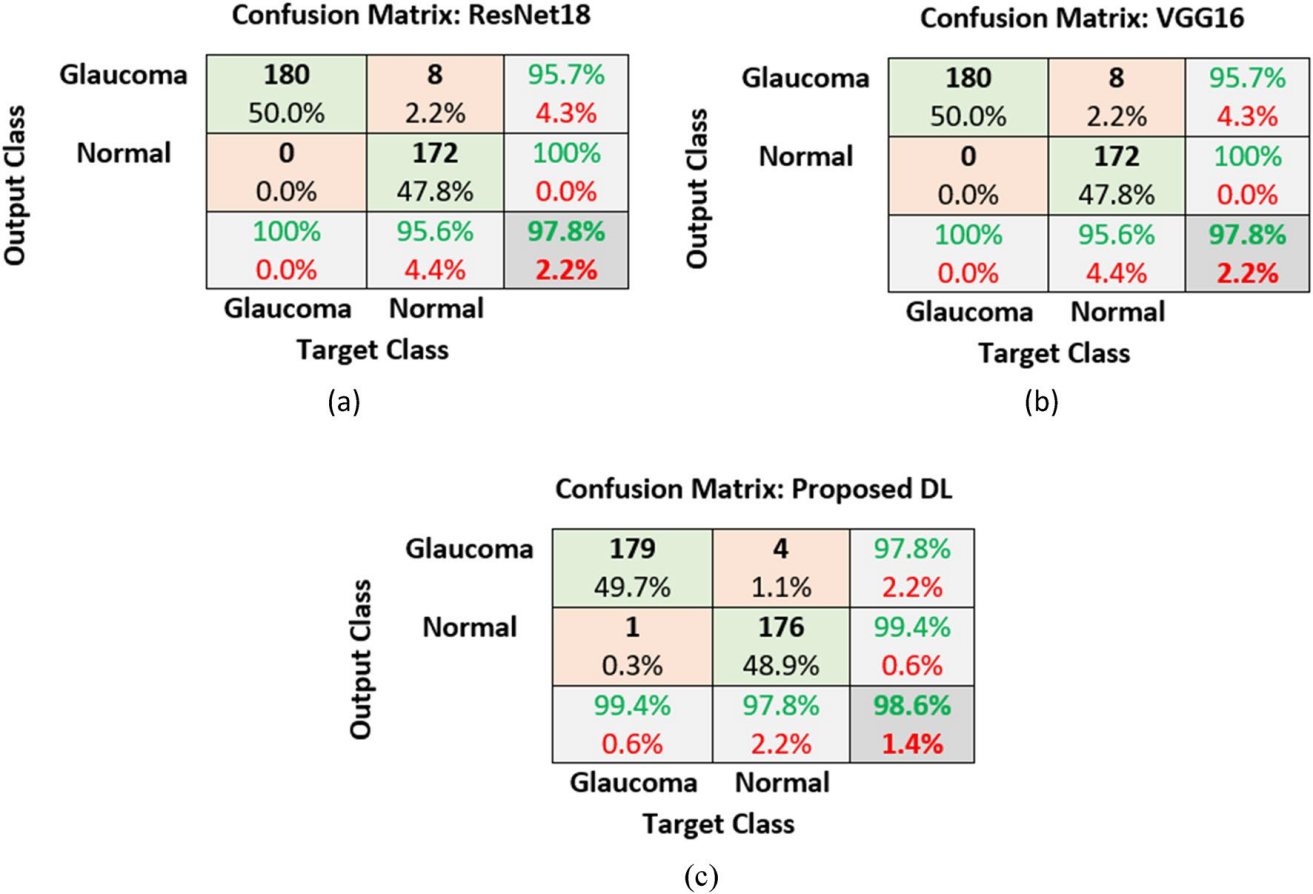
Confusion matrix obtained from (a) ResNet18 (b) VGG16 and (c) proposed DL model using five-fold cross-validation.

**Table 7:** Performance summary of ResNet18, VGG16 and proposed DL model, trained from the segmented OCT images.

DL architecture	AUC (mean ± SD)	Accuracy (mean ± SD)	Sensitivity (mean ± SD)	Specificity (mean ± SD)	Precision (mean ± SD)
ResNet18	0.99 ± 0.004	97.8 ± 2.12%	100 ± 0.0%	95.6 ± 4.21%	95.7 ± 3.77%
VGG16	0.99 ± 0.001	97.8 ± 1.60%	100 ± 0.0%	95.6 ± 3.16%	95.7 ± 2.94%
Proposed DL	0.99 ± 0.006	98.6 ± 1.50%	99.4 ± 1.25%	97.8 ± 2.34%	97.8 ± 2.22%

From the confusion matrix of Tables 6 and 7, it was demonstrated that the classification result of the ResNet18 and VGG16 model was similar with accuracy 97.8%, sensitivity 100%, specificity 95.6% and precision 95.7%. Our proposed DL model performed better than two pre-trained models with an AUC of 0.99, an accuracy of 98.6%, sensitivity 99.4%, and the specificity and glaucoma prediction precision of 97.8%. The AUC is 0.99 for the three DL model, which suggests that the maximum threshold values were able to successfully separate the two classes, i.e., glaucoma and normal, with the new segmented cup surface area. Among a total of 180 glaucoma images, 1 image has been misclassified as normal eyes, which are not subsequent images of one eye, i.e., among of 6 images per eye, only one image has been misclassified as a normal eye and the majority of the images per eye were correctly detected as glaucoma. A similar misclassification rate was also observed in the context of normal eyes.

### Heatmap visualization

**Figure 4:**
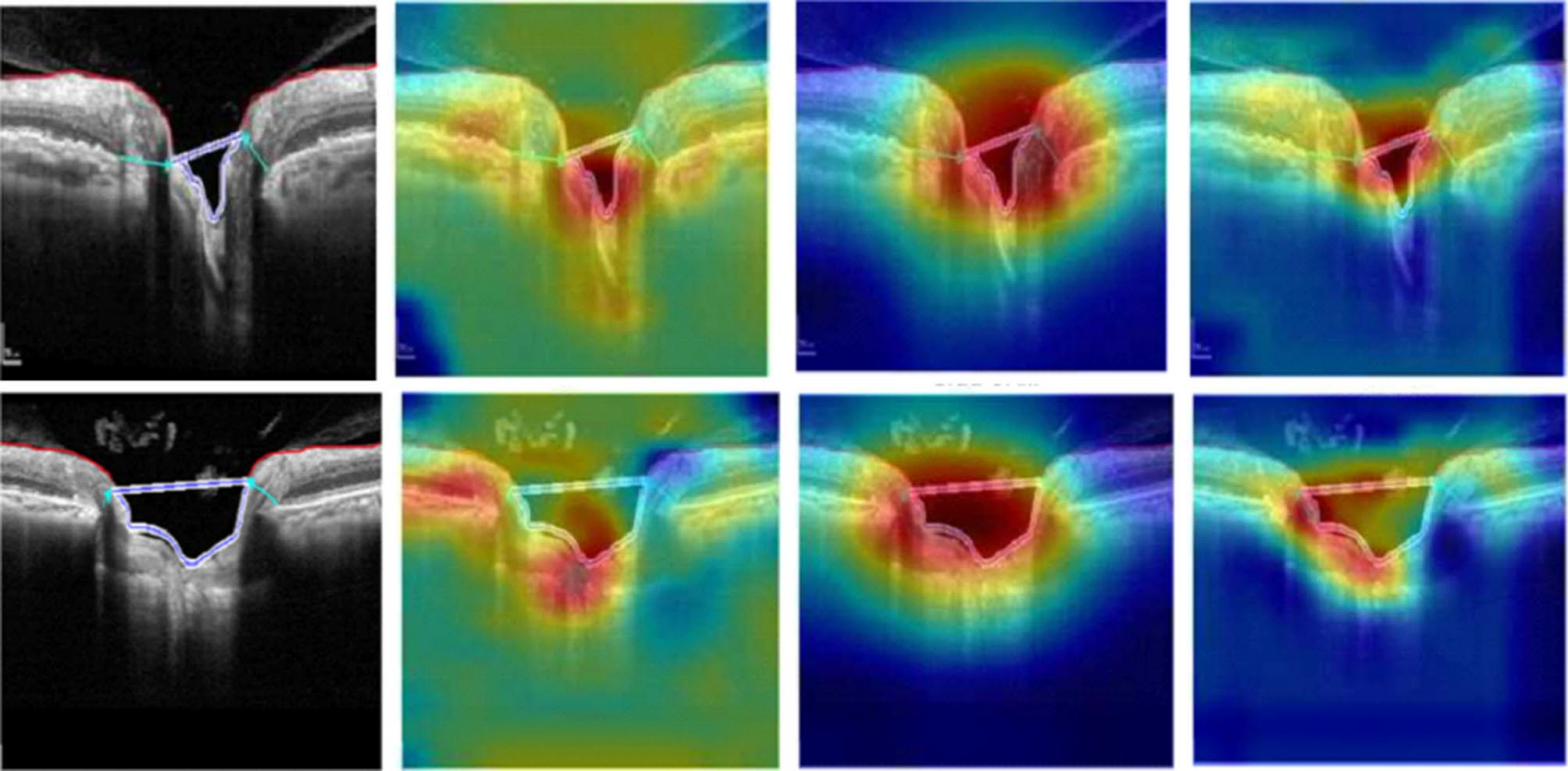
The GradCAM heatmaps for VGG16, ResNet18 and proposed DL model (left to right) obtained from segmented OCT images of glaucomatous eyes (left).

**Figure 5:**
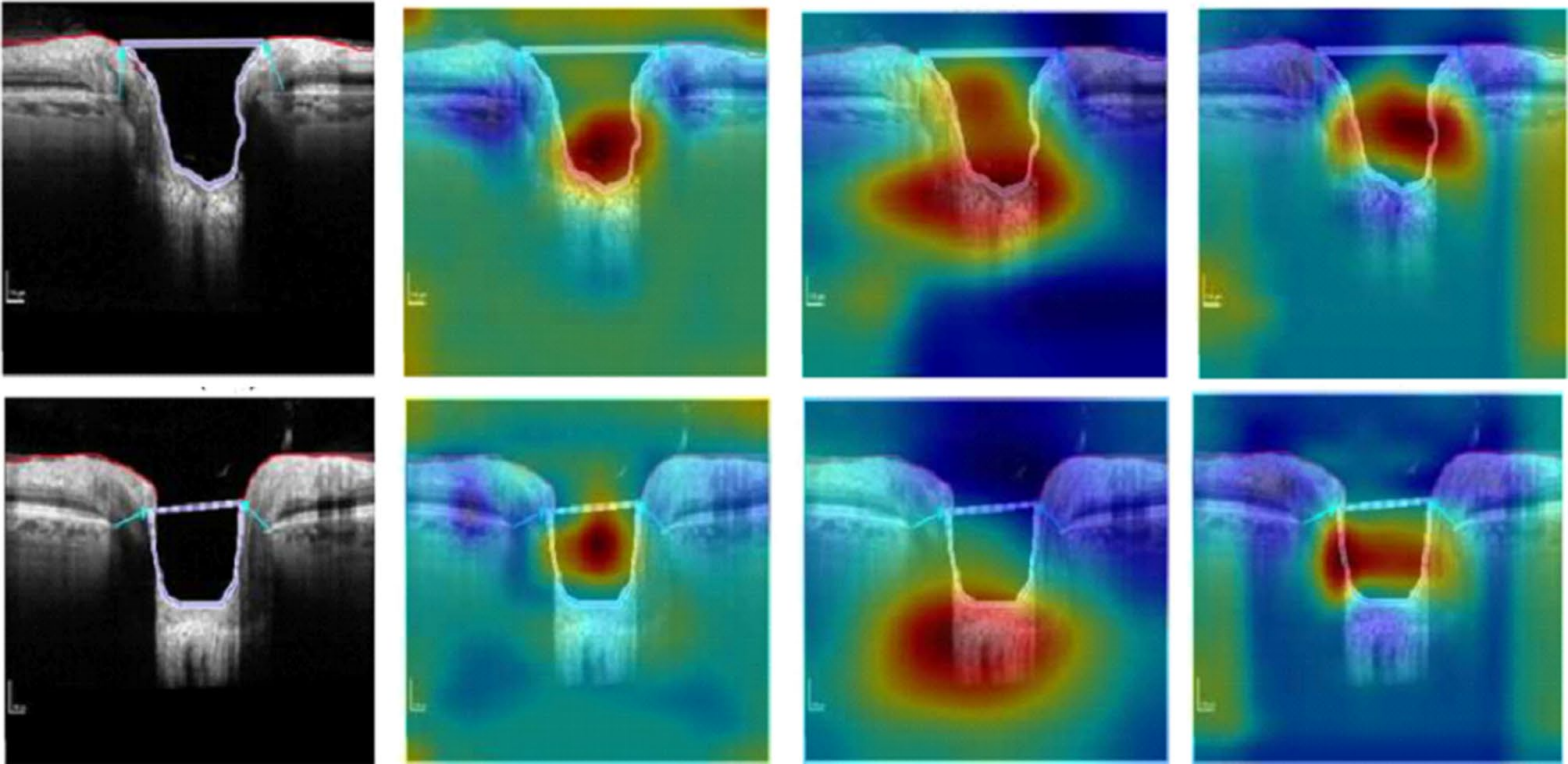
The GradCAM heatmaps for VGG16, ResNet18 and proposed DL model (left to right) obtained from segmented OCT images of normal eyes (left).

In Figs. 4 and 5, we show the heatmaps produced from Grad-CAM for a few randomly selected glaucomatous and normal OCT images to explain what features of the three models were considered to differentiate the two classes.

In Figs. 4 and 5, we can see the Grad-CAM produced deep red color inside or on the edges of the cup surfaces, which implied that the ONH cup surface area is able to differentiate between normal and glaucoma group by deep learning technique. Additionally, we can see the VGG16 and our proposed model accurately localized (deep red) the deformation of ONH cup surface for glaucoma images.

## Discussion

In this research, we collected diagnostic features from both normal and glaucomatous patients based on structural, functional, demographic, and risk factors and successfully optimized the features to detect glaucoma using ML algorithms. The statistical results are broadly in agreement with previous research^50^ that suggested that certain risk factors such as refractive error, IOP, family history and the presence of a thin cornea are not significant for glaucoma screening.

There are many studies of ML for automated glaucoma detection based on structural and non-structural features trained from fundus photography, OCT, and VF images^20–35^ mentioned in the literature. Glaucoma is a very complex disease, and we cannot rely on a single test, as there is insufficient information to make a diagnosis. This is the first study where optimal features were combined from structural, functional, demographic and risk factors to differentiate normal and glaucomatous eyes. In our previous study^34^, we have included all features, including age and VFI. The age and VFI both were found statistically significant between the two groups. In this study, we increased the data size and investigated how glaucoma features affected the wide range of the same age group as it is not only limited to older ages. Since VFI is calculated from the PD and MD values, we discarded VFI from our final features to reduce data processing requirements. Furthermore, we have also compared the DL results with and without VFI; the presence of VFI has no impact on the classification results. Moreover, early detection is essential as early glaucoma treatment can save or halt further vision loss. Therefore, in our study, we included a large number of glaucoma patients at early stages, and with the four features: RNFL, CDR, MD and PSD, both LR and DL model successfully able to detect glaucoma even at early stages. To the best of our knowledge, this is the first study where maximum glaucoma features were combined, analyzed, and trained using machine learning so that the optimal features could be able to detect glaucoma at early stages. The designed DL model showed better performance than the LR classifier on validation dataset. However, the LR and DL model performed similarly on the test dataset. Table 8 shows the comparison of the performance of our proposed DL model with previous studies of glaucoma detection using both structural and functional features. Our DL model showed promising performance on the validation dataset with an AUC of 0.98, 97% accuracy, 100% sensitivity and 96% specificity. For the test dataset, the accuracy 96%, sensitivity 100% and specificity 93%. Here, we particularly compare our study with previous findings based on combined structural and functional features. The primary aim of the comparison here is to demonstrate that our identified distinct features trained with deep learning has improved in automated glaucoma detection. Furthermore, an additional strength of the study is the inclusion of a wide range of ages, ranging from 30 to 85 in both groups, thereby resulting in a technique to optimize glaucoma features independent of subject age.

**Table 8:** Comparison of the performance of our proposed model with previous studies of glaucoma detection combining structural and functional features.

Studies	Method	Trained features	AUC	Accuracy (%)	Sensitivity (%)	Specificity (%)	No. of patients
Kim et al.^22^	RF model	Age, IOP, CCT, Average RNFL thickness, GHT, MD and PSD	0.98	98	98.3	97.5	202 healthy and 297 glaucoma
Brigatti et al.^23^	NN (Back propagation)	MD, corrected loss, variance, short term, fluctuation, CDR, rim area, cup volume, and RNFL height	–	88	90	84	54 healthy and 185 glaucoma
Bowd et al.^24^	RVM	OCT RNFL thickness measurements, MD, and PSD	0.85	–	81	72	69 healthy and 156 glaucoma
Grewal et al.^25^	ANN	RNFL parameters on OCT, cup area, vertical CDR, cup volume, MD, loss variance, and GDx- Variable Corneal Compensation (VCC) parameters	–	–	93.3	80	35 healthy and 35 glaucoma
Eliash et al.^51^	SVM	Horizontal integrated rim width (HIRW), rim area, HCDR, vertical CDR, Mean NFL, NFL inferior, NFL superior, NFL 6, NFL 7, NFL 11, and MD	0.98	96.6	97.9	92.5	47 healthy and 42 glaucoma
Proposed study	DL	MD, PSD, Average RNFL thickness and CDR	0.98	97% (validation data), 96% (test data)	100% (validation and test data)	96% (validation data), 93% (test data)	130 healthy and 125 glaucoma

Based on the proposed DL result of validation and test data, the sensitivity was 100%, which outperformed the accurate prediction of glaucoma even at the early stages. The specificity was 96% and reduced to 93% on the test data. A total of three: one validation and two test normal eyes were misdiagnosed as glaucomatous eye. The subjects were further investigated. First subject’s (age 60) MD, PSD, RNFL and CDR values were − 16.8, 8.1, 89.1 and 0.6, respectively, which could be predicted as suspect glaucoma or related to other diseases. The second subject (68) has higher PSD (4.92), and another subject (68) has lower RNFL thickness (79.65) and higher CDR (0.66), which suggests that there could be some ageing effect on MD, PSD, RNFL and CDR but further analytical study on a larger dataset is required. The result of the test data also indicates that the 2D data had shown promising performance on validation data for detecting glaucoma. Still, the false positive rate increased on test data which might be challenging for DL to separate disease from the normal. Therefore, in the pilot study, we used cross-sectional ONH OCT scans, which allowed us to assess the abnormalities of the anterior ONH and perform the quantitative measurement of the surface or contours if any damage results from glaucoma. We segmented and extracted a new structural feature, from the cross-sectional ONH OCT B-scans. We first segmented six radial B-scans of the SD-OCT ONH area and measured the ONH surface cup area for every scan, and finally calculated the mean area that revealed the actual changes of the superior and inferior region glaucomatous eyes. This demonstrated that the resultant mean cup area is significantly lower (p = 0.01) in glaucoma patients, which can be a useful marker for glaucoma diagnosis. We also developed a deep convolutional neural network to evaluate this new cup area feature for glaucoma detection.

The results of the DL trained with the segmented images showed good accuracy compared to two existing pre-trained models. Though VGG16, ResNet18 and our proposed model AUC were same for glaucoma detection, our DL model has a much simpler structure with less parameters and a shorter training duration than the two pre-trained models. Besides, our proposed DL model exhibited better performance compared to the results of previous studies^11–14^. Moreover, the GradCAM visualization also showed that the segmented cup surface is able to distinguish glaucoma from the normal group. The VGG16 and our proposed DL model accurately localized the affected region of glaucoma in the segmented OCT images. It is also noted that to the best of our knowledge, this is the first study using six cross-sectional OCT B-scan images trained together to increase the precision of the diagnostic assessments of ONH. The ONH cupping is a significant structural change for glaucoma patient. Based on previous literature^52–54^, the ONH cup is an important region of interest for the assessment of a glaucoma patient. Therefore, we conducted this pilot study to segment the ONH surface cup area and found it is significant between the two groups. This study suggested that segmentation and measurement of cross-sectional ONH cup area could be a novel clinical imaging feature to be added in the diagnosis of glaucoma. The proposed DL model using OCT ONH segmented images, glaucoma detection may be used as an effective screening tool for clinicians in glaucoma diagnostics.

This study has several limitations. First, our study mainly relied on the standard test for glaucoma diagnosis (e.g. Tonometry, Funduscopy, Perimetry/visual fields, Pachymetry and OCT scans). We were not able to include other advanced features like gonioscopy measurement, Retinal Ganglion Cell Layer (GCL) and GCL + Inner Plexiform Layer (IPL) thicknesses, OCT angiography [e.g. vessel density, blood flow index, flow-index, parapapillary deep-layer microvascular dropout (MvD)], and other risk factors like high myopia, high blood pressure, diabetes, Cardiovascular disease, previous eye surgery or injury associated with glaucoma^55–57^. Secondly, the study was conducted at the CFEH, Australia, where most of the patients were Caucasian and Asian; therefore, we could not explore the effect of ethnicity fully. In addition, we did not investigate the correlation of other diseases (like cataracts, diabetes, etc.) with glaucoma. These might have some effect on our glaucoma classification results. Finally, though the DL result of the pilot study with the new segmented feature is promising for glaucoma detection, but our study is limited to only the first 6 B-scans out of 24 radial B-scans collected by the Heidelberg SD-OCT. The segmentation of all 24 scans and measurement of the mean ONH cup area of total B-scans could make our study more reliable and clinically stable-which is an aim of future study.

Overall, the study of deep learning using four identical features and segmented ONH cup area as an input, individually has been found promising for glaucoma diagnosis. The cross-sectional segmented ONH cup area can be added as a new clinical feature for diagnosing glaucoma and might enhance the accuracy if combined with 2D data. In future, we aim to establish the findings in a large-scale clinical trial investigating the performance of deep learning by combining four features with the new segmented ONH cup surface area to detect glaucoma precisely regardless of different risk factors and ethnicity.
